# The *Drosophila melanogaster methuselah* Gene: A Novel Gene with Ancient Functions

**DOI:** 10.1371/journal.pone.0063747

**Published:** 2013-05-16

**Authors:** Ana Rita Araújo, Micael Reis, Helder Rocha, Bruno Aguiar, Ramiro Morales-Hojas, Sandra Macedo-Ribeiro, Nuno A. Fonseca, David Reboiro-Jato, Miguel Reboiro-Jato, Florentino Fdez-Riverola, Cristina P. Vieira, Jorge Vieira

**Affiliations:** 1 Instituto de Biologia Molecular e Celular (IBMC), Universidade do Porto, Porto, Portugal; 2 Center of Research in Advanced Computing Systems (CRACS-INESC Porto), Universidade do Porto, Porto, Portugal; 3 EMBL-European Bioinformatics Institute, Hinxton, Cambridge, United Kingdom; 4 Departamento de Informática, Universidade de Vigo, Ourense, Spain; Fred Hutchinson Cancer Research Center, United States of America

## Abstract

The *Drosophila melanogaster* G protein-coupled receptor gene, *methuselah* (*mth),* has been described as a novel gene that is less than 10 million years old. Nevertheless, it shows a highly specific expression pattern in embryos, larvae, and adults, and has been implicated in larval development, stress resistance, and in the setting of adult lifespan, among others. Although *mth* belongs to a gene subfamily with 16 members in *D. melanogaster*, there is no evidence for functional redundancy in this subfamily. Therefore, it is surprising that a novel gene influences so many traits. Here, we explore the alternative hypothesis that *mth* is an old gene. Under this hypothesis, in species distantly related to *D. melanogaster*, there should be a gene with features similar to those of *mth*. By performing detailed phylogenetic, synteny, protein structure, and gene expression analyses we show that the *D. virilis GJ12490* gene is the orthologous of *mth* in species distantly related to *D. melanogaster*. We also show that, in *D. americana* (a species of the *virilis* group of *Drosophila*), a common amino acid polymorphism at the *GJ12490* orthologous gene is significantly associated with developmental time, size, and lifespan differences. Our results imply that *GJ12490* orthologous genes are candidates for developmental time and lifespan differences in *Drosophila* in general.

## Introduction

G protein-coupled receptors (GPCRs) are a large and important group of receptor proteins involved in signal transduction. They can be classified into five large families, namely Glutamate-like receptors, Rhodopsin-like receptors, Adhesion, Frizzled, and Secretin-like receptors [1], and are known to participate in a variety of biological processes, from light transduction to hormone signaling and development (see for instance, [1]–[3]). Therefore, it is not surprising that GPCRs are present in both protostomes and deuterostomes (see for instance, [1]–[3]). Nevertheless, within the *secretin* family there is one subfamily that has been reported as being insect-specific, the *methuselah* subfamily (see for instance, [4]).

Secretin receptors are characterized by long N-terminal domains that tend to recognize peptide ligands, such as, hormones and neuropeptides [5], [6], and in insects are known to play a role in important biological processes, such as, the setting of the endogenous circadian clocks that affect behavior and reproduction [7], regulation of fluid and ion secretion [8], as well as, stress response and longevity [9]. Although the functional role of the *mth* subfamily is largely unknown, they likely play an important role during embryo and larval development [4]. Surprisingly, the number of *mth*-like genes varies widely in different species from 16 in *D. melanogaster* [4] to only two in *Bombix mori* [10].

The *mth*-like gene family is named after the *Drosophila melanogaster methuselah* (*mth*) gene [4]. Mth shows an N-terminal Mth ectodomain and a C-terminal seven transmembrane (7 tm) domain [11]. An important feature of the Mth ectodomain (only found in members of the *mth* subfamily) is the presence of 10 cysteine residues that form five disulfide bonds [11]. *mth* is significantly expressed during gastrulation, in the imaginal disc progenitor cells, in the third-instar central nervous system, and larval imaginal discs [4] (http://flybase.org/). Moreover, in adult flies, this gene is significantly expressed in the crop, Malpighian tubules, heart and spermatheca (http://flybase.org/). This gene has been shown to be required in the presynaptic motor neuron to acutely upregulate neurotransmitter exocytosis at larval glutamatergic neuromuscular junctions [12].

Although *mth* is clearly involved in embryo and larval development [4], [12], most studies on this gene are related to the setting of adult lifespan. Mutants that code for a truncated version of the Mth protein have an extended lifespan [9], and the same effect is achieved by the use of Mth inhibitors [13]. Recently, it has been shown that reduced expression of *mth* in insulin-producing cells (IPCs) of the fly brain is sufficient to extend life [14]. Interestingly, overexpression of *mth* in these cells has similar phenotypic effects to reduced expression [14]. Reduced Mth signaling also inhibits insulin secretion from the IPCs, and therefore, the longevity enhancement might be through reduced insulin/IGF signaling [14]. In *D. melanogaster*, there are significant associations between naturally occurring Mth amino acid polymorphisms and lifespan differences [15], [16].

Besides extended lifespan, *mth* mutants also show enhanced resistance to stress [9], and reduced expression of *mth* in IPCs of the fly brain is sufficient to enhance oxidative stress resistance [14]. Moreover *mth* mutants display a higher wing-beat frequency and coordinated visuomotor entrainment to motion during simulated flight [17], as well as no slowing of the rate of stem cell division with age [18]. Therefore, it has been suggested that *mth* mutants not only have delayed chronological aging but also enhanced sensorimotor abilities critical to survival during early and middle, but not late life [17]. It should be noted, however, that *mth* mutants do not show enhanced behavioral performance in all tasks [19], [20]. Moreover, Baldal *et al.* [21] showed that the lifespan enhancing effect of the *mth* mutants is completely abolished by mating, and these mutants also show trade-off effects between extended lifespan and reproductive output [21], [22]. Therefore, non-functional *mth* alleles are unlikely to increase in frequency in natural populations.

Changes in *mth* transcript levels are thought to be related to the observed lifespan variation [9], [12], [15], [16], [23], [24]. Nevertheless, *mth* also exhibits a significant pattern of adaptive amino acid divergence among *D. melanogaster*, *D. simulans* and *D. yakuba* [15], [16], indicating that amino acid changes have been positively selected. The adaptive amino acid changes appear to be localized to those regions of the protein that modulate signal transduction [16].

Patel *et al.* [4] reported that *mth* is a novel gene (less than 10 million years old) that is present in species of the *melanogaster* subgroup only. Zhang *et al.* [25] reported that genes expressed in many tissues tend to be old. Therefore, it is surprising that a developmental gene with a highly specific expression pattern in embryos, larvae, and adults, and with so many pleiotropic effects, could have evolved recently. It should be noted that there is no evidence for functional redundancy in the *mth* gene subfamily [4]. We have thus explored the possibility that *mth* is an old gene. If this is the case, then in species distantly related to *D. melanogaster*, there should be a gene with features similar to those shown by *mth*. Here, we perform detailed phylogenetic, synteny, protein structure, and gene expression analyses that show that the *D. virilis GJ12490* gene is the orthologous gene of *mth* in species distantly related to *D. melanogaster*. In *D. americana* (a species of the *virilis* group of *Drosophila*), a common amino acid polymorphism at the *GJ12490* orthologous gene is here shown to be significantly associated with developmental time, size, and lifespan.

## Materials and Methods

### Gene Sequences

tBlastn searches were performed at FlyBase (http://flybase.org/) using as a query the *D. melanogaster* Methuselah protein (AAF47379.2), and the 12 publicly available and annotated *Drosophila* genomes. All retrieved sequences are annotated in FlyBase as *mth*-like sequences. Accession numbers are given in the figures showing the phylogenetic results (see results section). The same procedure was used to identify one *mth*-like sequence in *Daphnia pulex* (see results).

### Phylogenetic Analyses

Phylogenetic analyses were performed using the ADOPS pipeline [26]. When ADOPS is used, nucleotide sequences are first translated and aligned using the amino-acid alignment as a guide. In order to determine how the choice of a given alignment algorithm influences the phylogenetic reconstruction, three separate analyses were performed using the ClustalW2, MUSCLE and T-Coffee alignment algorithms as implemented in T-Coffee [27]. It should be noted that when using ADOPS, only codons with a support value above two are used for phylogenetic reconstruction. Alignments of the *mth*-like copies of *D. melanogaster* resulting from MUSCLE and ClustalW2 were tested for nucleotide substitution saturation by plotting the observed number of transitions and transversions against the genetic distance (F84) as implemented in DAMBE v. 5.3.15 [28] (Fig. S1).

Bayesian trees were obtained using MrBayes 3.1.2 [29] as implemented in the ADOPS pipeline. The model of sequence evolution implemented in the analyses was the GTR, allowing for among-site rate variation and a proportion of invariable sites. Third codon positions were allowed to have a gamma distribution shape parameter different from that of first and second codon positions. Two independent runs of 2,000,000 generations with four chains each (one cold and three heated chains) were set up. The average standard deviation of split frequencies was always about 0.01 and the potential scale reduction factor for every parameter about 1.00 showing that convergence has been achieved. Trees were sampled every 100 th generation and the first 5000 samples were discarded (burn-in). The remaining trees were used to compute the Bayesian posterior probabilities of each clade of the consensus tree. A BI (Bayesian inference) analysis was performed with each alignment (obtained with ClustalW2 and MUSCLE) as input. The sequence of the related gene *cirl* was used as outgroup, as in Patel *et al.* ([4]; see also the results section). The two alternative BI phylogenetic trees obtained with each alignment were compared using the Approximately Unbiased (AU) test [30] as implemented in the program CONSEL v0.1j [31].

### Detection of Protein Domains

Protein domains were detected using the Conserved Domains tool at NCBI (http://www.ncbi.nlm.nih.gov/Structure/cdd/wrpsb.cgi; [32]).

### Homology Modelling

Mth ectodomain theoretical models were obtained using the I-TASSER [33] server (zhanglab.ccmb.med.umich.edu/I-TASSER/). The highest TM-score value that is obtained when performing a structural alignment using the TM-align algorithm (zhanglab.ccmb.med.umich.edu/TM-align/) was used to build a distance matrix by subtracting these values from one. The resulting matrix was used to build an UPGMA tree using Mega5 [34].

### Gene Expression Analyses

Total RNA was isolated from adults and L2 larvae from *D. americana* and *D. virilis* using TRIzol Reagent (Invitrogen) according to the manufacturer’s instructions and treated with DNase I (RNase-Free) (Ambion). cDNA was synthesized by reverse transcription with SuperScript III First-Strand Synthesis SuperMix for qRT-PCR (Invitrogen). cDNAs were amplified using the PCR conditions and the specific primers shown on Table S1. When performing the PCR, no template controls and reactions where no reverse transcriptase was added, were performed in order to confirm the absence of genomic DNA contamination (data not shown). The presence or absence of gene expression was observed by agarose gel electrophoresis.

### F2 Association Experiment

The *D. americana* F2 association study using strains H5 and W11, described in detail in [35], was used to test for associations between variation at the *GJ12490* orthologous gene and developmental time, abdominal size and longevity. The first trait to be measured was developmental time. For this purpose, each of the 83 second generation crosses (F1) were transferred to new flasks every day in order to obtain the precise period of time between oviposition and adult emergence. The resulting F2 males were then individually collected. When F2 males were 10 days old (young adult flies), individual chill-coma recovery times were measured at +25°C after four hours of cold exposure at 0°C. Flies must be able to stand up on their legs in order to be considered completely recovered. Individual photographs were taken when individuals were 20 days old, using a stereomicroscope Nikon ZMS 1500 H. The resulting JPG files were saved with a resolution of 1600×200 pixels. Relative abdominal size was estimated by counting the number of pixels in the picture that correspond to this structure, using Adobe Photoshop H. The flies were then transferred to new vials and kept until they died, in order to measure lifespan. Only males were used in order to avoid potential confounding effects caused by differences between sexes for the traits being studied (see for instance [37]). 453 F2 *D. americana* males showing extreme phenotypes (after excluding the individuals that show at least two phenotypic values in the second third of the distribution; the phenotypes that were considered are developmental time, chill-coma recovery time, abdominal size and lifespan), that are the descendants of three F0 H5 x W11 crosses (named crosses A, B and C), were selected out of 975 individuals.

In this experiment isofemale rather than isogenic strains were used. Therefore, we sequenced a short fragment of the Mth ectodomain identified in *GJ12490* gene in order to check for segregating polymorphisms within strains. The F0 individuals used were screened by direct sequencing of the amplification products obtained with primers Mth29_nsyn_F (TGCTAACACTGCTATTTCTA) and Mth29_nsyn_R (GCGTGATGACCGTTTTGT) using standard PCR conditions with an annealing temperature of 52°C. Amplification products were purified using Gel Extraction kit from QIAGEN (Izasa Portugal, Lda.). Sequencing was performed using ABI PRISM Big Dye cycle-sequencing kit version 1.1 (Perkin Elmer, CA, USA) and primers Mth29_nsyn_F and Mth29nsyn_seqR (CGTGCGTTCATTGCTGTC). Sequencing runs were performed by STABVIDA (Lisbon, Portugal). Given the evidence for heterozigosity in cross A, only crosses B and C were used. The F0 individuals used in crosses B and C, in the sequenced region, differ only at one putative highly conserved N-glycosilation amino acid site of the Mth ectodomain of the protein encoded by the *GJ12490* orthologous gene. Since there was no restriction enzyme available to type the difference at the N-glycosilation amino acid site, a molecular marker in the close vicinity was used to follow this difference in the F2 individuals. The genomic region with 697 bp was amplified using standard PCR conditions and primers Mth_29_F (GTTCTTTCCGAGCAGCAA) and Mth_29_R (CAGAGCACACAGCAGAGC) with an annealing temperature of 53°C. The PCR products were then digested with the restriction enzyme *Sau*3AI and typed as 0 (undigested), 1 (completely digested) and 0/1 (heterozygous).

All statistical tests and summary statistics were computed using the software SPSS Statistics 17.0 (SPSS Inc., Chicago, Illinois). Linear regression analyses (including a constant) were performed in order to estimate the percentage of variation in developmental time, lifespan and size that can be explained by the common amino acid polymorphism at the *D. americana GJ12490* orthologous gene. This may be an overestimate since, as noted above, we used only males showing extreme phenotypes for developmental time, chill-coma recovery time, abdominal size and lifespan.

### *Drosophila americana* Wild-caught Individuals

In order to determine the frequency and distribution in natural populations of the N/S polymorphism at the protein encoded by the *GJ12490* orthologous gene, 12 wild-caught *D. americana* male individuals from Fremont (Nebraska, July, 2008) and eight individuals from Saint Francisville (Louisiana, July, 2010) were used, respectively. These individuals were screened by direct sequencing of the amplification products obtained with primers Mth29_nsyn_F (TGCTAACACTGCTATTTCTA) and Mth29_nsyn_R (GCGTGATGACCGTTTTGT) using standard PCR conditions with an annealing temperature of 52°C. Amplification products were purified using Gel Extraction kit from QIAGEN (Izasa Portugal, Lda.). Sequencing was performed using ABI PRISM Big Dye cycle-sequencing kit version 1.1 (Perkin Elmer, CA, USA) and the sequencing primers Mth29_nsyn_F and Mth29nsyn_seqR (CGTGCGTTCATTGCTGTC). Sequencing runs were performed by STABVIDA (Lisbon, Portugal).

## Results

### Evolutionary History of the *mth*-like Gene Family in *Drosophila*

In order to address the question of whether *mth* is only present in the *melanogaster* subgroup as suggested by Patel *et al.* [4], detailed phylogenetic analyses are here performed using the sequences obtained from the search of the publicly available and annotated *Drosophila* genomes (http://flybase.org/). Given the level of polytomy present in the deeper branches of the tree obtained by Patel *et al.* [4], it is not possible to determine which copy is the ancestral *mth*-like gene (it could be either *mthl1*, *mthl5*, *mthl14,* or *mthl15* (*CG31720*)). Mthl1, Mthl5, and Mthl14 do not show a recognizable Mth ectodomain or the 10 cysteines typical of the Mth ectodomain (results not shown).

We first inferred the relationship of all *D. melanogaster mth*-like genes, the *D. melanogaster cirl* gene (the *secretin* gene used by Patel *et al.* [4] to root their tree), and the *Daphnia pulex gi321478728* gene. The *Daphnia* sequence shows a 7 tm domain but does not show a recognizable Mth ectodomain. Five out of the 10 cysteine residues typical of the Mth ectodomain are, however, conserved. Four out of the 10 typical cysteine residues are not conserved, but there are cysteine residues in the close proximity (less than ten amino acid residues away) in the *Daphnia* sequence. When performing Blastp, as implemented in FlyBase (http://flybase.org/), using the *Daphnia* putative Mth-like ectodomain region sequence as a query, there are significant hits (expect value smaller than 0.05) with *Drosophila* proteins encoded by members of the *mth* gene family only. It should be noted that the *Daphnia pulex gi321478728* gene could be miss-annotated since there is an extra 153 amino acid block at the N terminal region that has no correspondence in Mth-like proteins (data not shown).

**Figure 1:**
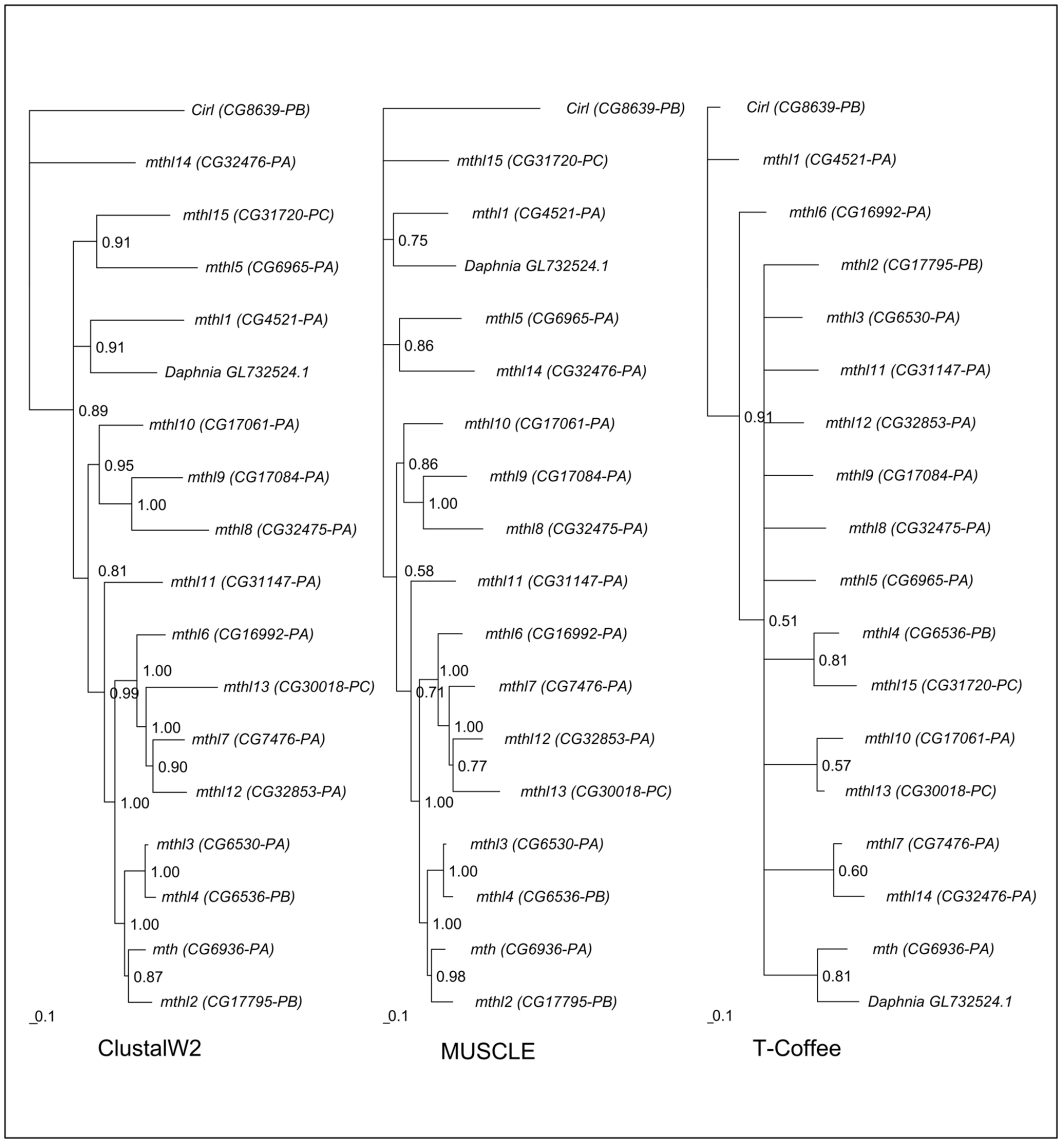
Phylogenetic relationships of the *D.* melanogaster mth-like genes, the *D. melanogaster cirl* gene and the *Daphnia pulex gi321478728* gene, using three different alignment methods, as implemented in ADOPS [26]. Values near the nodes are posterior credibility values.

*mth*-like genes have been reported from insects only [3], [4]. Therefore, the expectation was that the gene sequence from *Daphnia pulex* would be placed in the phylogeny as the sister lineage to the *D. melanogaster mth* subfamily clade. This is, however, not the case, and this conclusion is not dependent on the alignment algorithm that is used (Fig. 1). When the ClustalW2 and the MUSCLE alignment algorithm are used, the *Daphnia pulex* sequence clusters with the *D. melanogaster mthl1* gene sequence. Therefore, the *Daphnia* sequence seems to be the orthologous of the *mthl1* gene. It should be noted that, like the *Daphnia* sequence, the *D. melanogaster* protein encoded by *mthl1* does not have a recognizable Mth ectodomain, and does not show conservation of all 10 typical cysteine residues. It should also be noted that, in all the Mth-like proteins without a recognizable Mth ectodomain this region can be aligned with that of Mth, although it seems to be divergent enough to be identified as a Mth ectodomain (data not shown). The phylogenetic trees shown in Fig. 1 are rooted using the *D. melanogaster cirl* gene as outgroup, since the alternative rooting (rooting the trees using the *Daphnia pulex* sequence) would imply that the *D. melanogaster cirl* gene is a *mth*-like gene.

The phylogenetic inferences here made (Fig. 1) show that the choice of the alignment algorithm influences the conclusion on which one is the oldest *mth*-like gene. It should be noted that the levels of substitution saturation were high in the alignments (Fig. S1), thus, inferences from the phylogenetic analyses should be taken with care. However, some inferences can be made in combination with other lines of evidence. The sister relationship of *mthl1* with a gene in a Crustacean species, and the structural homology analyses here performed (see below), suggest that *mthl1* is the ancestral *mth*-like gene copy in *Drosophila*. The phylogenetic analysis of the ClustalW2 alignment, however, places *mthl14* as the sister gene copy to a clade comprising all the other *mth*-like genes (and including also *Daphnia*’s gene). On the other hand, when the MUSCLE algorithm is used, the phylogeny shows a basal polytomy including *mthl5*, *mthl15* (*CG31720*), *mthl14* and *mthl1.* Since it is not possible to infer which one is the most ancestral gene, in all subsequent Bayesian phylogenetic analyses we arbitrarily rooted the trees using the *mthl14* gene, although this gene may not be the oldest one. We avoided using the *cirl* gene as the outgroup in the subsequent analyses, since, being highly divergent from *mth*-like genes (less than 30% amino acid identities), it would introduce many alignment gaps (the Cirl protein is about three times larger than Mth-like proteins), reducing the number of positions that could be analyzed, and it would increase phylogenetic noise.

**Figure 2:**
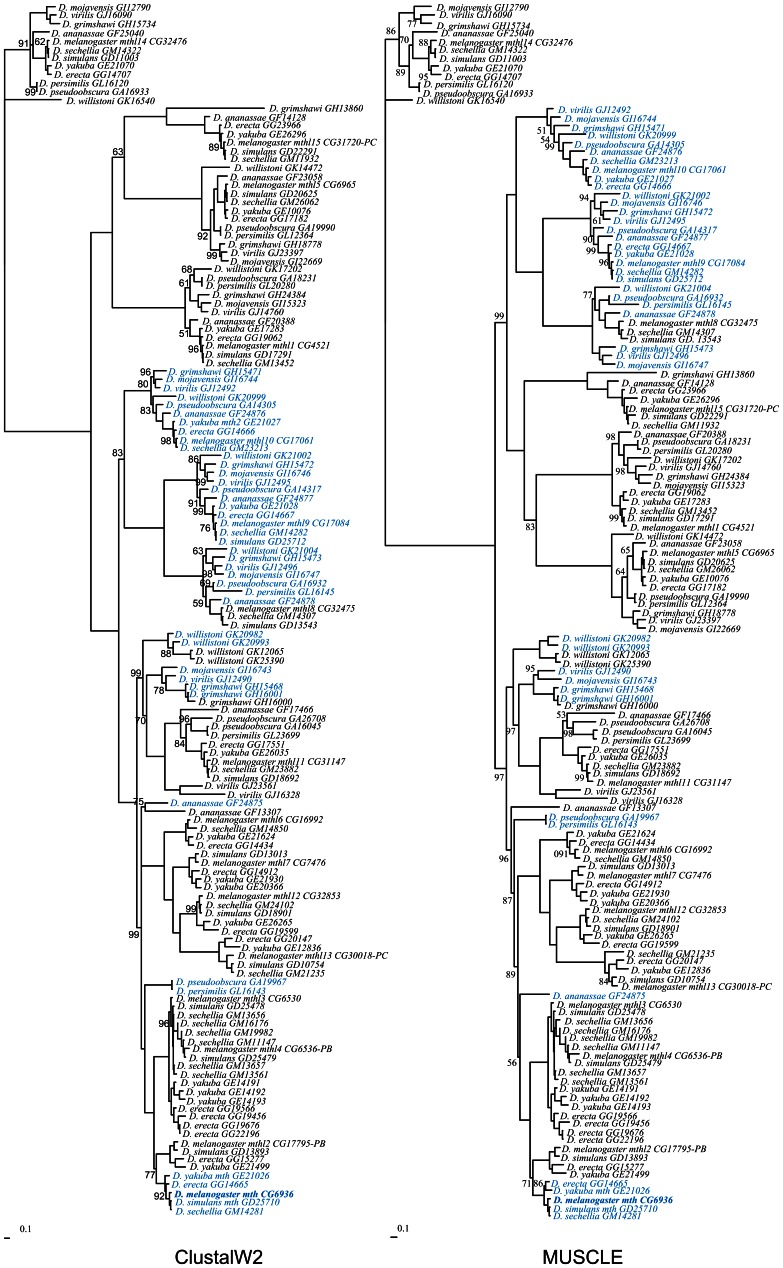
*mth*-like DNA sequence relationships inferred using a Bayesian approach. Values near the nodes are posterior credibility values. When there is no value near the node the posterior credibility value is 100%. When more than one transcript is available for the same gene, transcript PA has been used, unless otherwise stated. Sequences marked in light blue correspond to the genes that belong to the *mth* cluster.

The inferred phylogenetic relationships obtained with two different alignments (ClustalW2 and MUSCLE) of 143 *Drosophila mth*-like gene sequences retrieved from the 12 annotated *Drosophila* genomes, are shown in Fig. 2. It should be noted that 12 additional sequences coding for proteins shorter than 250 amino acids (about half the size of Mth) were not used. The T-Coffee algorithm failed to produce an alignment that could be used for phylogenetic analyses (data not shown).

In agreement with the results of Patel *et al.* [4], species of the *melanogaster* subgroup show more *mth-*like genes (15–22) than the other *Drosophila* species analyzed (9–11; Table 1; the 12 sequences not included in the phylogenetic analyzes have been taken into consideration for these calculations). In the *melanogaster* subgroup lineage, there are many recent gene duplication events of such genes, such as, the *D. melanogaster* genes *mth*, *mthl2*, *mthl3*, *mthl4*, *mthl6*, *mthl7*, *mthl12*, and *mthl13*.

**Table 1:** *mth*-like gene number in *Drosophila* species.

Species	Subgenus	Group	Subgroup	*mth*-like gene number
*D. melanogaster*	*Sophophora*	*melanogaster*	*melanogaster*	16
*D. simulans*	*Sophophora*	*melanogaster*	*melanogaster*	15
*D. sechellia*	*Sophophora*	*melanogaster*	*melanogaster*	22
*D. erecta*	*Sophophora*	*melanogaster*	*melanogaster*	17
*D. yakuba*	*Sophophora*	*melanogaster*	*melanogaster*	18
*D. ananassae*	*Sophophora*	*melanogaster*		10
*D. pseudoobscura*	*Sophophora*	*obscura*		9
*D. persimilis*	*Sophophora*	*obscura*		9
*D. willistoni*	*Sophophora*	*willistoni*		10
*D. mojavensis*	*Drosophila*	*repleta*		9
*D. virilis*	*Drosophila*	*virilis*		9
*D. grimshawi*	*Drosophila*	*hawaiian drosophila*		11

**Figure 3:**
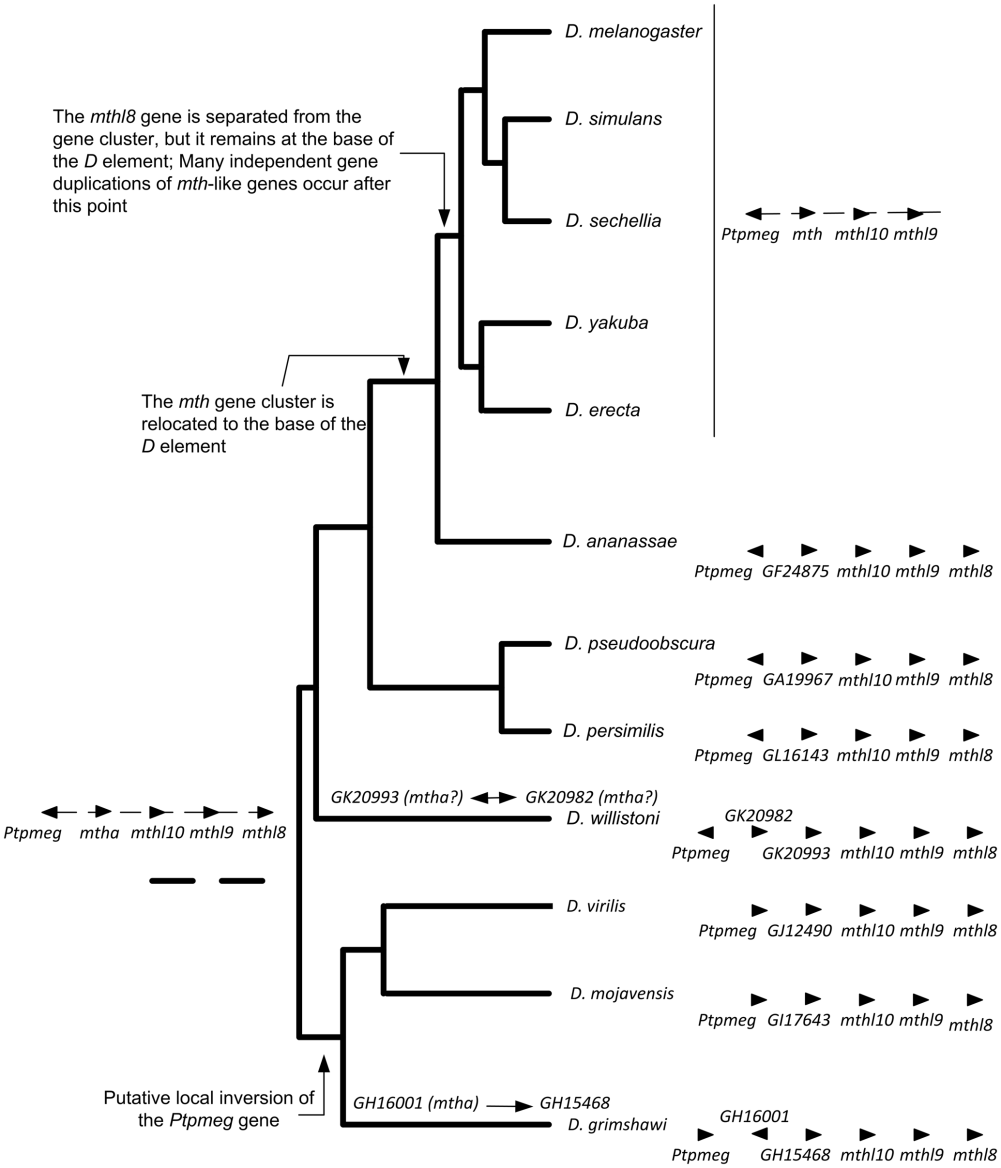
Suggested evolutionary hypothesis for the *mth* gene cluster.

The phylogenetic inferences here made, as well as, the synteny information that is available at FlyBase (http://flybase.org/; see also Fig. 3) implies that in distantly related *Drosophila* species, the *GJ12490* orthologous gene is the best candidate for performing a function similar to that of the *D. melanogaster mth* gene. Indeed, the *GJ12490* gene is (together with *GJ23561* and *GJ16328*) the one that is most closely related to *mth.* Moreover, *GJ12490* is the neighbor of the *Ptpmeg* and *mthl10* genes as it is *mth*. It should be noted, however, that the relative orientation of *Ptpmeg* gene is different in species from the *Drosophila* and *Sophophora* subgenus. Moreover, the GJ12490 protein shows all the typical features of Mth, including the presence of a recognizable Mth ectodomain with 10 conserved cysteines, and a 7 tm domain. It should be noted that in FlyBase (http://flybase.org/), the *D. virilis GJ12490* gene is miss-annotated, having four extra exons 5′ of the start of the Mth ectodomain that belongs to a *Drosophila* gene from the odorant receptor family (data not shown). All *D. virilis mth*-like genes are expressed both in L2 larvae and adults, showing that they are not pseudogenes (data not shown). These genes are also expressed both in *D. melanogaster* larvae and adults with the exception of *mthl11* (http://flybase.org/). In *D. americana*, a species closely related to *D. virilis*, all *mth*-like genes are also expressed both in L2 larvae and adults (data not shown).

### Structural Analysis of the *GJ12490* Mth Ectodomain

**Figure 4:**
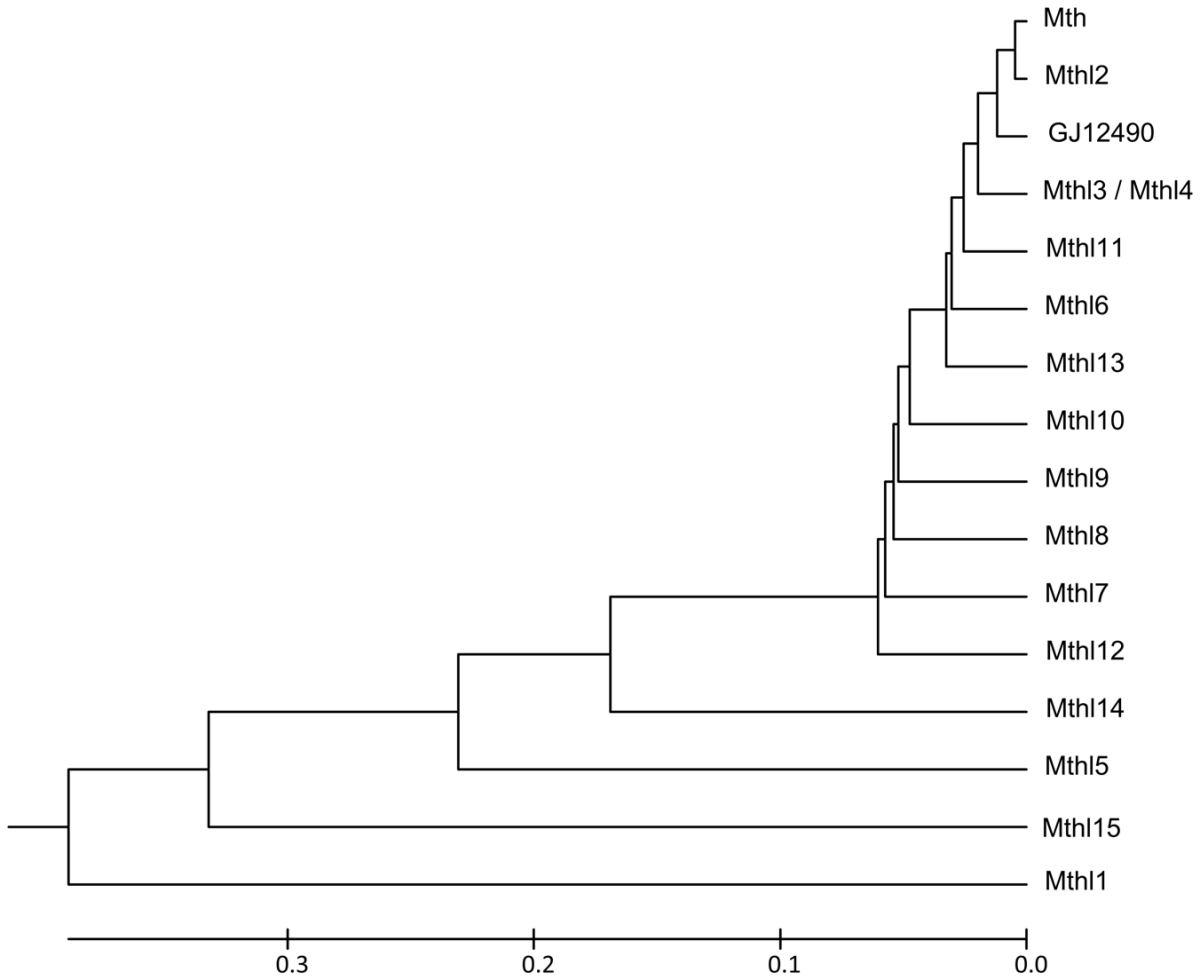
UPGMA tree showing the overall similarity of the Mth ectodomain of the proteins encoded by *D.* melanogaster mth-like genes and the protein encoded by the *D. virilis GJ12490* gene. The values used to build the distance matrix were obtained by subtracting to 1 the lower TM-score value that is obtained when performing a structural alignment using the TM-align algorithm (zhanglab.ccmb.med.umich.edu/TM-align/).

**Figure 5:**
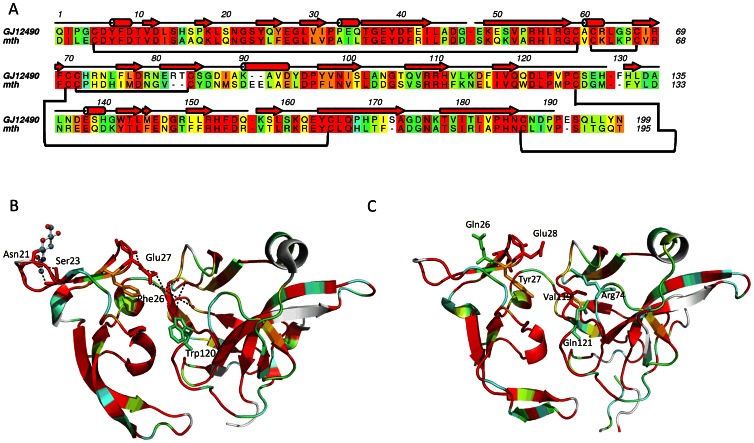
Structural features of the Mth ectodomain. A) The amino acid sequence alignment of the extracellular ectodomain of *D. melanogaster* Mth and *D. virilis* GJ12490. The alignment was performed with ClustalW2, and colored with Aline [40]. The residues are colored according to a residue conservation scale (residues that are identical or similar among the sequences are given a colored background; red: identical residues; orange to blue: scale of conservation of amino acid properties in each alignment column; white: dissimilar residues). The numbers above the alignment refer to GJ12490 and the secondary structure to Mth (red cylinders, α-helices; red arrows, β-sheets). The lines below the sequences correspond to the cysteine residues involved in each disulfide bond. B) Cartoon representation of *D. melanogaster* Mth extracellular ectodomain 3D structure (PDB ID code 1FJR), highlighting the glycosylated Asn21 and the network of hydrogen bonds (dashed black lines) that a) stabilize the β2–β3 hairpin centered on Ser23 and b) interconnect this hairpin (through Glu27) to the central pocket and to the exposed Trp120. The color-coded scales for each amino acid correspond to residue conservation as shown in the sequence alignment. Water molecules are represented as red spheres. C) Cartoon representation of GJ12490 theoretical model colored as B).

In order to obtain further evidence that the *GJ12490* and *mth* could be performing a similar function, we obtained homology models of the structure of the Mth ectodomain of all proteins encoded by *D. melanogaster mth*-like genes, as well as the structure of the Mth ectodomain of the protein encoded by gene *GJ12490*. It is clear that the *D. virilis* GJ12490 Mth ectodomain is highly similar to that of the *D. melanogaster* Mth protein (Fig. 4). The pattern of disulphide bonds that stabilize the 3D structure of the extracellular ectodomain is strictly conserved. The putative glycosylation site (Asn21, using as a reference the 1FJR accession) is maintained. Although the role of glycosylation remains unknown, from the structural point of view the covalently bound N-acetylglucosamine in *D. melanogaster* ectodomain seems to play a role in stabilization of the β2–β3 hairpin by forming hydrogen bonds with the conserved Ser at position 23 (Fig. 5). The conserved Glu27 and Phe26 stacking at the tip of this hairpin are at the interface between the D1 and the D2D3 subdomains, and outline a putative ligand-binding region. Glu27 also forms part of a water-mediated hydrogen-bond network interconnecting the two subdomains, which also involves the non-conserved and exposed Trp120 (Fig. 5). Therefore one might hypothesize that removal of the carbohydrate at position 21 is likely to increase the flexibility of this hairpin and therefore impact on the interface between the two D1 and D2D3 subdomains, altering the size and conformation of a putative ligand binding pocket. The predicted size of the pocket, where the ligand putatively binds is similar in GJ12490 (753 Å^3^) and Mth (746 Å^3^) proteins (Fig. 5).

### Naturally Occurring Variation at the *D. americana GJ12490* Orthologous Gene is Associated with Developmental Time and Abdominal Size Differences

**Figure 6:**
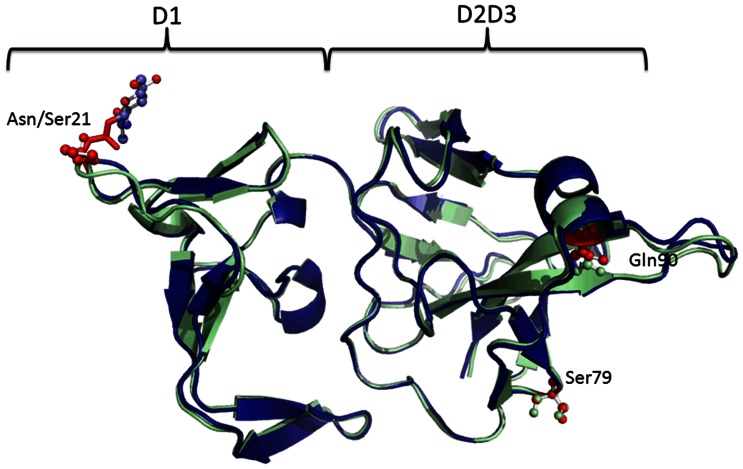
The predicted structure of the *D.* americana Mth ectodomain (the S variant is shown in blue and the N variant in green). The orange and red sticks are the *D. virilis* structurally equivalent residues.

**Figure 7:**
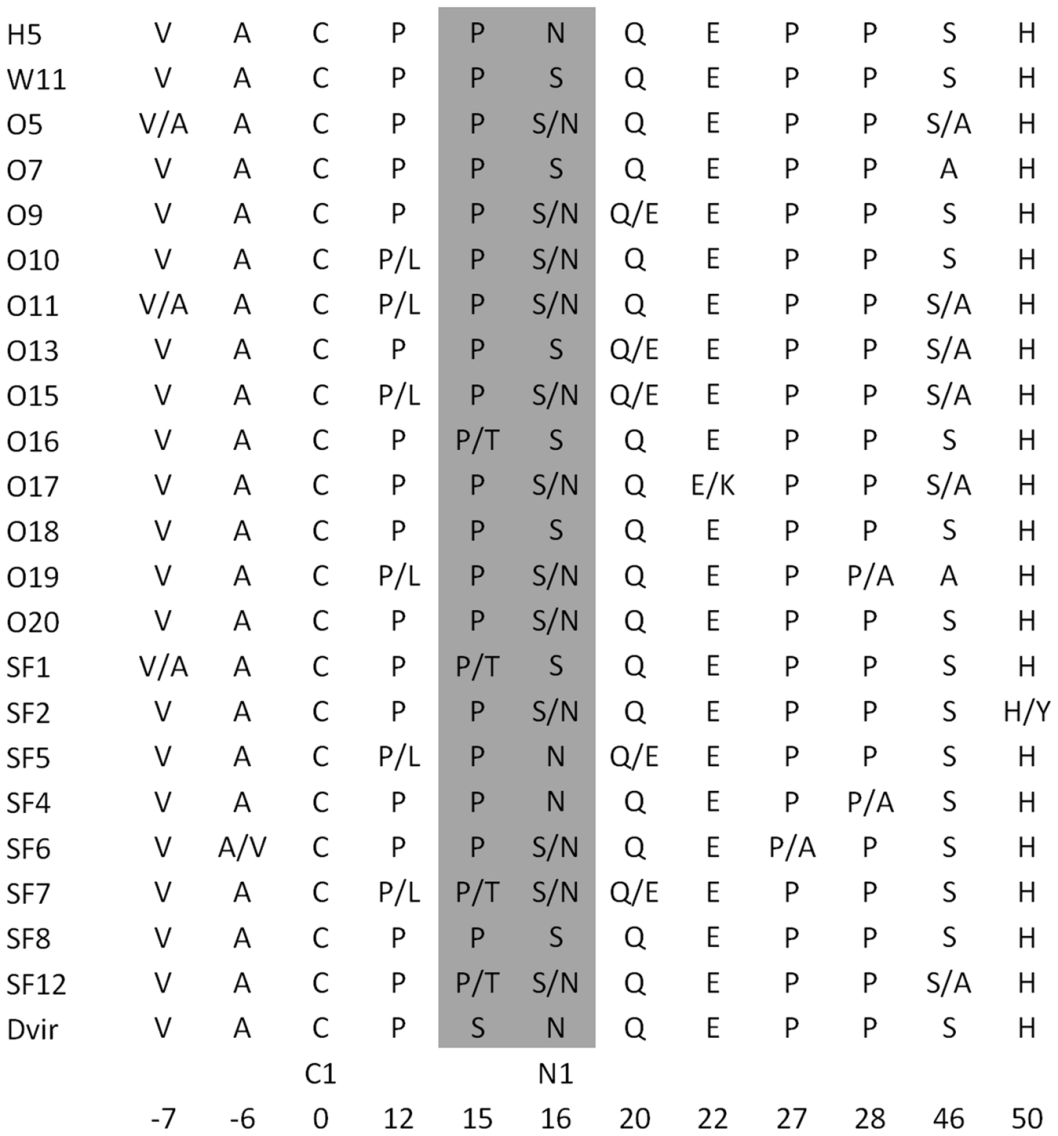
Amino acid variation at the *D. americana mth*-like GJ12490 protein. The amino acid replacement that is segregating in the F2 association experiment is highlighted in grey. C1– the first typical cysteine; N1– putative N-glycosylation site. Amino acid positions are given relative to the first typical cysteine.

The *D. melanogaster mth* gene has been shown to be a developmental gene [4], [12] although there is no evidence that it affects developmental time. Nevertheless, if gene *GJ12490* is playing a role similar to that of *mth* during development, then variability at this gene could be associated with developmental time differences from egg to adult. In order to address this issue, we used *D. americana*, a species closely related to *D. virilis* (the common ancestor of the two species lived about 4.1 million years ago; [36] and an F2 association design (see Material and Methods)). In the F2 association experiment, the *GJ12490* variant 0 (not digested; assayed by digesting with *Sau*3AI the 697 bp *mth-*like *GJ12490* gene amplification product; see Material and Methods) goes with an N at position +16 relative to the first cysteine of the Mth ectodomain, while variant 1 (digested) goes with an S. The substitution of an N by an S destroys the putative highly conserved N-glycosylation site, and thus, this amino acid substitution could influence the stability of the hairpin harboring the mutation. However the presence of a Pro residue at position 20 within the tight turn of the β2–β3 hairpin will likely counterbalance the effect of the disappearance of the consensus glycosylation site (Figs. 6 and 7). Therefore, the N/S polymorphism does not seem to influence the capacity to bind a ligand. Only individuals not having a Pro at the preceding amino acid are predicted to be non-functional. Nevertheless, the non-glycosylation of this site may affect other aspects such as folding, maturation and/or intracellular trafficking of the protein.

Significant associations are found between genotype (N/N, N/S and S/S) and developmental time (Non-parametric Kruskal-Wallis Test; P<0.001). The average and standard deviation for genotypic classes N/N, S/S and N/S is 15.6±2.0 days (N = 54), 16.7±1.8 days (N = 83), and 15.7±1.9 days (N = 155), respectively. Significant differences are detected, using Non-parametric Mann-Whitney tests, between genotypes N/N and S/S (P<0.005), and S/S and N/S (P<0.001), but not between genotypes N/N and N/S (P>0.05). The significant differences remain significant after the sequential Bonferroni correction for multiple comparisons (P<0.05). Therefore, allele S seems to be recessive over allele N and to be associated with slow developmental time. Under this model, the N/S polymorphism explains 5.5% (P<0.001) of the variability observed in the F2 association experiment regarding developmental time (Pearson correlation coefficient = 0.235; P<0.001; the 95% lower and upper confidence limits for the correlation coefficient, determined by bootstrap (1000 samples), are 0.119 and 0.346, respectively). Individuals with the S/S genotype take on average 6.6% longer to develop than the remaining individuals.

The flies of the F2 association experiment have been phenotyped for abdominal size, as well. It is conceivable that developmental time differences give rise to adult size differences. Therefore, we looked for an association between the N/S polymorphism and abdominal size, and found a significant association (Non-parametric Kruskal-Wallis Test; P<0.005). The average and standard deviation for genotypic classes N/N, S/S and N/S is 1.02±0.13 relative units (N = 54), 0.95±0.14 relative units (N = 83), and 1.00±0.14 relative units (N = 155), respectively. Significant differences are detected, using Non-parametric Mann-Whitney tests, between genotypes N/N and S/S (P<0.005), and S/S and N/S (P<0.005), but not between genotypes N/N and N/S (P>0.05). The significant differences remain significant after the sequential Bonferroni correction for multiple comparisons (P<0.05). Therefore, the S allele seems to be recessive over the N allele, and to be associated with small abdominal size. Individuals with the S/S genotype are on average 5.8% smaller than individuals carrying at least one N allele.

Under this model, variation at gene *GJ12490* explains 3.1% (P<0.005) of the variability observed in the F2 association experiment regarding abdominal size (Pearson correlation = 0.175; P<0.005; the 95% lower and upper confidence limits for the correlation coefficient, determined by bootstrap (1000 samples), are 0.062 and 0.285, respectively).

### Evidence that Naturally Occurring Variation at Gene *GJ12490* Affects Lifespan

In *D. melanogaster*, variability at the *mth* gene has been reported to influence lifespan (see introduction). In order to gather further evidence that gene *GJ12490* could be playing the same role as the *mth* gene, we looked for an association between the N/S polymorphism (see above) and lifespan. A significant association between genotype and lifespan was found (Non-parametric Kruskal-Wallis Test; P<0.005). The average and standard deviation for genotypic classes N/N, S/S and N/S is 49.4±20.0 days (N = 54), 59.2±19.8 days; (N = 83), and 60.6±21.1 days (N = 155). There are no significant differences regarding lifespan when genotypes S/S and N/S are compared (Non-parametric Mann-Whitney Test; P>0.05) but significant changes are observed when comparing genotypes N/N and N/S (Non-parametric Mann-Whitney Test; P<0.001), and when comparing genotypes N/N and S/S (Non-parametric Mann-Whitney Test; P<0.01). These results are significant after applying the sequential Bonferroni correction for multiple testing (P<0.05). Therefore, N/N genotype seems to be associated with short lifespan. Individuals with genotypes S/S and N/S show a 21.7% average increase in lifespan in comparison with individuals with genotype N/N. Under the assumption that the N allele is recessive over the S allele, the N/S polymorphism explains 3.8% (Pearson correlation coefficient = 0.200; P<0.005; the 95% lower and upper confidence limits for the correlation coefficient, determined by bootstrap (1000 samples), are 0.078 and 0.310, respectively) of the lifespan variability observed in the F2 association experiment.

### Frequency of the N and S Variant in Natural Populations

In order to determine the frequency and distribution in natural populations of the N/S polymorphism at the protein encoded by the *GJ12490* orthologous gene, 12 wild-caught *D. americana* male individuals from Fremont (Nebraska) and eight individuals from Saint Francisville (Louisiana) were used (Fig. 7). The derived S allele (by comparison with *D. virilis*) is at a high frequency in both the northern (66.6%) and southern (50.0%) populations, and thus, may help explain a significant amount of the variability regarding developmental time, abdominal size and lifespan in natural populations.

## Discussion

It has been argued that *mth* is a novel gene that is only present in species of the *melanogaster* subgroup [4]. It is surprising that a developmental gene that is expressed in embryos, larvae and adults, and that has many pleiotropic effects is of recent origin (see introduction), and therefore, here, we tested the alternative hypothesis that *mth* is an old gene. Under this hypothesis, it should be possible to find a gene with features similar to those presented by *mth* in *Drosophila* species distantly related to *D. melanogaster*. Here, we show that the neighbors of *mth* and *D. virilis GJ12490* are the same, that phylogenetic analyzes indicate that this gene is one of the most closely related to *mth*, that the predicted structure of the Mth ectodomain of the protein encoded by gene *GJ12490* is very similar to the structure of the Mth ectodomain of the *mth* gene, and that the two genes have similar broad expression patterns. Moreover, in *D. americana* (a species of the *virilis* group of *Drosophila*), there is a common amino acid polymorphism at the protein encoded by the *GJ12490* orthologous gene that shows a strong association with developmental time, size and lifespan in an F2 association experiment, although in this case we cannot rule out the possibility that the association is caused by the presence of a neighbor gene. The association with developmental time is expected since *mth* is clearly a developmental gene [4]. On the other hand, naturally occurring amino acid variation at the *D. melanogaster mth* gene was previously associated with lifespan differences [15], [16], and in our F2 association cross, individuals with genotypes S/S and S/N show a 21.7% average increase in lifespan in comparison with individuals with genotype N/N. This number is similar to the about 30% lifespan extension described for *D. melanogaster mth* mutant individuals [9]. Therefore, it is likely that *mth* and *GJ12490* orthologous genes are playing similar roles. This is an important observation because it implies that *GJ12490* orthologous genes could be candidates for explaining developmental time and lifespan differences in *Drosophila* in general. It should be noted that, despite of all similarities, Mth and the protein encoded by gene GJ12490 show less than 50% amino acid identity.

While addressing the above hypothesis we found a *mthl1* gene in the Crustacean species *Daphnia pulex*. Therefore, the *mth* subfamily is not insect specific as previously reported. The *mth* subfamily must thus be older than 420 million years, the age of the separation of Insects and Crustaceans [37]. It is not possible to infer the early evolution of the *mth*-like gene family since the conclusions are highly dependent on the alignment algorithm that is used, and it is not possible to determine which alignment algorithm works best in general (see the discussion in [38]). Such a dependence on the alignment algorithm used has been reported in other cases involving old gene families as well [38], [39] and shows the need to use multiple alignment algorithms when studying such cases, which can be easily done when using ADOPS [26]. As suggested by Patel et al. [4], subfunctionalization may be an important feature of the evolution of the *mth* gene subfamily. Detailed functional comparative studies are now needed in order to understand whether *mth*-like genes acquired novel functionalities after the recent gene duplications events that are detected in the *melanogaster* subgroup lineage.

## Supporting Information

Figure S1 Transitions (blue diamonds) and transversions (red squares) versus genetic distance plots showing the level of nucleotide substitution saturation at different codon positions (all positions (1); 1^st^ and 2^nd^ codon positions (2) and 3^rd^ codon positions (3)) using the two different alignments (ClustalW2 (A) and MUSCLE (B)).
(TIF)

Table S1 PCR conditions used to address *mth*-like genes' expression in *D. virilis* and *D. americana*.
(XLSX)

## References

[1] Fredriksson R, Lagerström MC, Lundin L-G, Schiöth HB (2003) The G-protein-coupled receptors in the human genome form five main families. Phylogenetic analysis, paralogon groups, and fingerprints. Mol Pharmacol 63: 1256–1272. 12761335 10.1124/mol.63.6.1256

[2] Brody T, Cravchik A (2000) *Drosophila melanogaster* G protein–coupled receptors. J Cell Biol 150: F83–F88. 10908591 10.1083/jcb.150.2.f83 PMC2180217

[3] Nordström KJV, Lagerström MC, Wallér LMJ, Fredriksson R, Schiöth HB (2009) The *Secretin* GPCRs descended from the family of *Adhesion* GPCRs. Mol Biol Evol 26: 71–84. 18845549 10.1093/molbev/msn228

[4] Patel MV, Hallal DA, Jones JW, Bronner DN, Zein R, et al (2012) Dramatic expansion and developmental expression diversification of the *methuselah* gene family during recent *Drosophila* evolution. J Exp Zool B Mol Dev Evol 318: 368–387. 22711569 10.1002/jez.b.22453

[5] Cardoso JCR, Clark MS, Viera FA, Bridge PD, Gilles A, et al (2005) The secretin G-protein-coupled receptor family: teleost receptors. J Mol Endocrinol 34: 753–765. 15956345 10.1677/jme.1.01730

[6] Cardoso J, Vieira F, Gomes A, Power D (2010) The serendipitous origin of chordate secretin peptide family members. BMC Evol Biol 10: 135. 20459630 10.1186/1471-2148-10-135 PMC2880984

[7] Mertens I, Husson SJ, Janssen T, Lindemans M, Schoofs L (2007) PACAP and PDF signaling in the regulation of mammalian and insect circadian rhythms. Peptides 28: 1775–1783. 17586087 10.1016/j.peptides.2007.05.005

[8] Reagan JD (1994) Expression cloning of an insect diuretic hormone receptor. A member of the calcitonin/secretin receptor family. J Biol Chem 269: 9–12. 8276884

[9] Lin Y-J, Seroude L, Benzer S (1998) Extended Life-Span and Stress Resistance in the *Drosophila* Mutant *methuselah* . Science 282: 943–946. 9794765 10.1126/science.282.5390.943

[10] Fan Y, Sun P, Wang Y, He X, Deng X, et al (2010) The G protein-coupled receptors in the silkworm, *Bombyx mori* . Insect Biochem Mol Biol 40: 581–591. 20685615 10.1016/j.ibmb.2010.05.005

[11] West AP, Llamas LL, Snow PM, Benzer S, Bjorkman PJ (2001) Crystal structure of the ectodomain of Methuselah, a *Drosophila* G protein-coupled receptor associated with extended lifespan. Proc Natl Acad Sci U S A 98: 3744–3749. 11274391 10.1073/pnas.051625298 PMC31123

[12] Song W, Ranjan R, Dawson-Scully K, Bronk P, Marin L, et al (2002) Presynaptic Regulation of Neurotransmission in *Drosophila* by the G Protein-Coupled Receptor Methuselah. Neuron 36: 105–119. 12367510 10.1016/s0896-6273(02)00932-7

[13] Ja WW, West AP, Delker SL, Bjorkman PJ, Benzer S, et al (2007) Extension of *Drosophila melanogaster* life span with a GPCR peptide inhibitor. Nat Chem Biol 3: 415–419. 17546039 10.1038/nchembio.2007.2 PMC2803097

[14] Gimenez LED, Ghildyal P, Fischer KE, Hu H, Ja WW, et al (2013) Modulation of methuselah expression targeted to *Drosophila* insulin-producing cells extends life and enhances oxidative stress resistance. Aging Cell 12: 121–129. 23121290 10.1111/acel.12027 PMC3552032

[15] Schmidt PS, Duvernell DD, Eanes WF (2000) Adaptive evolution of a candidate gene for aging in *Drosophila* . Proc Natl Acad Sci U S A 97: 10861–10865. 10995474 10.1073/pnas.190338897 PMC27114

[16] Duvernell DD, Schmidt PS, Eanes WF (2003) Clines and adaptive evolution in the *methuselah* gene region in *Drosophila melanogaster* . Mol Ecol 12: 1277–1285. 12694290 10.1046/j.1365-294x.2003.01841.x

[17] Petrosyan A, Hsieh IH, Saberi K (2007) Age-dependent stability of sensorimotor functions in the life-extended *Drosophila* mutant *Methuselah* . Behav Genet 37: 585–594. 17534708 10.1007/s10519-007-9159-y

[18] Wallenfang MR, Nayak R, DiNardo S (2006) Dynamics of the male germline stem cell population during aging of *Drosophila melanogaster* . Aging Cell 5: 297–304. 16800845 10.1111/j.1474-9726.2006.00221.x

[19] Cook-Wiens E, Grotewiel MS (2002) Dissociation between functional senescence and oxidative stress resistance in *Drosophila* . Exp Gerontol 37: 1347–1357. 12559404 10.1016/s0531-5565(02)00096-7

[20] Martinez VG, Javadi CS, Ngo E, Ngo L, Lagow RD, et al (2007) Age-related changes in climbing behavior and neural circuit physiology in *Drosophila* . Dev Neurobiol 67: 778–791. 17443824 10.1002/dneu.20388

[21] Baldal EA, Brakefield PM, Zwaan BJ, Hughes K (2006) Multitrait evolution in lines of *Drosophila melanogaster* selected for increased starvation resistance: the role of metabolic rate and implications for the evolution of longevity. Evolution 60: 1435–1444. 16929660

[22] Mockett RJ, Sohal RS (2006) Temperature-dependent trade-offs between longevity and fertility in the *Drosophila* mutant, *methuselah* . Exp Gerontol 41: 566–573. 16677788 10.1016/j.exger.2006.03.015

[23] Pletcher SD, Macdonald SJ, Marguerie R, Certa U, Stearns SC, et al (2002) Genome-wide transcript profiles in aging and calorically restricted *Drosophila melanogaster* . Curr Biol 12: 712–723. 12007414 10.1016/s0960-9822(02)00808-4

[24] Kim H, Kim J, Lee Y, Yang J, Han K (2006) Transcriptional regulation of the *methuselah* gene by dorsal protein in *Drosophila melanogaster* . Mol Cells 21: 261–268. 16682822

[25] Zhang YE, Landback P, Vibranovski M, Long M (2012) New genes expressed in human brains: Implications for annotating evolving genomes. Bioessays 34: 982–991. 23001763 10.1002/bies.201200008

[26] Reboiro-Jato D, Reboiro-Jato M, Fdez-Riverola F, Vieira CP, Fonseca NA, et al (2012) ADOPS - Automatic detection of positively selected sites. J Integr Bioinform 9: 200. 22829571 10.2390/biecoll-jib-2012-200

[27] Notredame C, Higgins DG, Heringa J (2000) T-Coffee: A novel method for fast and accurate multiple sequence alignment. J Mol Biol 302: 205–217. 10964570 10.1006/jmbi.2000.4042

[28] Xia X, Xie Z (2001) DAMBE: software package for data analysis in molecular biology and evolution. J Hered 92: 371–373. 11535656 10.1093/jhered/92.4.371

[29] Ronquist F, Huelsenbeck JP (2003) MrBayes 3: Bayesian phylogenetic inference under mixed models. Bioinformatics 19: 1572–1574. 12912839 10.1093/bioinformatics/btg180

[30] Shimodaira H (2002) An approximately unbiased test of phylogenetic tree selection. Syst Biol 51: 492–508. 12079646 10.1080/10635150290069913

[31] Shimodaira H, Hasegawa M (2001) CONSEL: for assessing the confidence of phylogenetic tree selection. Bioinformatics 17: 1246–1247. 11751242 10.1093/bioinformatics/17.12.1246

[32] Marchler-Bauer A, Lu S, Anderson JB, Chitsaz F, Derbyshire MK, et al (2011) CDD: a conserved domain database for the functional annotation of proteins. Nucleic Acids Res 39: D225–D229. 21109532 10.1093/nar/gkq1189 PMC3013737

[33] Roy A, Kucukural A, Zhang Y (2010) I-TASSER: a unified platform for automated protein structure and function prediction. Nat Protoc 5: 725–738. 20360767 10.1038/nprot.2010.5 PMC2849174

[34] Tamura K, Peterson D, Peterson N, Stecher G, Nei M, et al (2011) MEGA5: molecular evolutionary genetics analysis using maximum likelihood, evolutionary distance, and maximum parsimony methods. Mol Biol Evol 28: 2731–2739. 21546353 10.1093/molbev/msr121 PMC3203626

[35] Reis M, Vieira CP, Morales-Hojas R, Aguiar B, Rocha H, et al (2011) A comparative study of the short term cold resistance response in distantly related *Drosophila* species: The role of *regucalcin* and *Frost* . PLoS ONE 6: e25520. 21991316 10.1371/journal.pone.0025520 PMC3184994

[36] Morales-Hojas R, Reis M, Vieira CP, Vieira J (2011) Resolving the phylogenetic relationships and evolutionary history of the *Drosophila virilis* group using multilocus data. Mol Phylogen Evol 60: 249–258. 10.1016/j.ympev.2011.04.022 21571080

[37] Gaunt MW, Miles MA (2002) An insect molecular clock dates the origin of the insects and accords with palaeontological and biogeographic landmarks. Mol Biol Evol 19: 748–761. 11961108 10.1093/oxfordjournals.molbev.a004133

[38] Santos JS, Fonseca NA, Vieira CP, Vieira J, Casares F (2010) Phylogeny of the teashirt-related zinc finger (tshz) gene family and analysis of the developmental expression of *tshz2* and *tshz3b* in the zebrafish. Dev Dyn 239: 1010–1018. 20108322 10.1002/dvdy.22228

[39] Vieira J, Fonseca NA, Vieira CP (2009) *RNase*-based gametophytic self-incompatibility evolution: questioning the hypothesis of multiple independent recruitments of the *S*-pollen gene. J Mol Evol 69: 32–41. 19495553 10.1007/s00239-009-9249-y

[40] Bond CS, Schuttelkopf AW (2009) *ALINE*: a WYSIWYG protein-sequence alignment editor for publication-quality alignments. Acta Crystallogr Sect D Biol Crystallogr 65: 510–512. 19390156 10.1107/S0907444909007835

