# Supplementary figures and images for: The Drosophila melanogaster methuselah Gene: A Novel Gene with Ancient Functions
Source: PLoS One. 2013 May 16;8(5):e63747. doi: 10.1371/journal.pone.0063747 (PMC3655951; doi:10.1371/journal.pone.0063747)

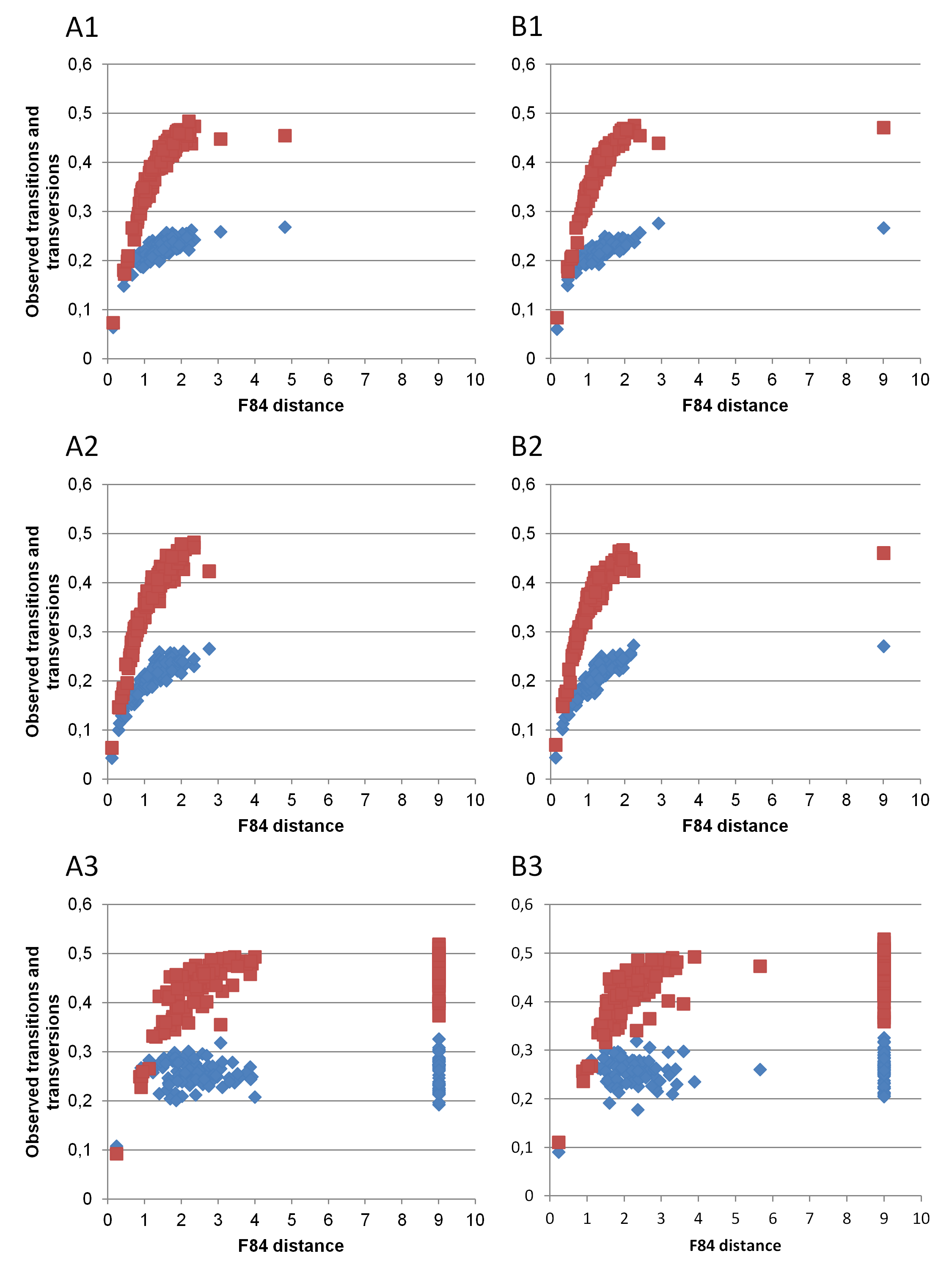

Supplement: Figure S1 — Transitions (blue diamonds) and transversions (red squares) versus genetic distance plots showing the level of nucleotide substitution saturation at different codon positions (all positions (1); 1st and 2nd codon positions (2) and 3rd codon positions (3)) using the two different alignments (ClustalW2 (A) and MUSCLE (B)). (TIF) [file pone.0063747.s001.tif]
